# Outcomes in participants with ventilated nosocomial pneumonia and organ failure treated with ceftolozane/tazobactam versus meropenem: a subset analysis of the phase 3, randomized, controlled ASPECT-NP trial

**DOI:** 10.1186/s13613-022-01084-8

**Published:** 2023-02-11

**Authors:** Ignacio Martin-Loeches, Andrew F. Shorr, Richard G. Wunderink, Marin H. Kollef, Jean-François Timsit, Brian Yu, Jennifer A. Huntington, Erin Jensen, Christopher J. Bruno

**Affiliations:** 1 St James’ Hospital, Dublin, Ireland; 2 Universitat de Barcelona, IDIBAPS, CIBERes, Barcelona, Spain; 3 Georgetown University, Washington, DC USA; 4 Northwestern University Feinberg School of Medicine, Chicago, IL USA; 5 Washington University School of Medicine, St. Louis, MO USA; 6 APHP Medical and Infectious Diseases ICU, Bichat Hospital Université Paris Cité, Paris, France; 7 Merck & Co., Inc., Rahway, NJ USA

**Keywords:** ASPECT-NP, Gram-negative, Nosocomial, Pneumonia, *Pseudomonas*, Shock, SOFA

## Abstract

**Background:**

The pivotal ASPECT-NP trial showed ceftolozane/tazobactam was non-inferior to meropenem for the treatment of ventilated hospital-acquired/ventilator-associated bacterial pneumonia (vHABP/VABP). Here, we evaluated treatment outcomes by degree of respiratory or cardiovascular dysfunction.

**Methods:**

This was a subset analysis of data from ASPECT-NP, a randomized, double-blind, non-inferiority trial (ClinicalTrials.gov NCT02070757). Adults with vHABP/VABP were randomized 1:1 to 3 g ceftolozane/tazobactam or 1 g meropenem every 8 h for 8–14 days. Outcomes in participants with a baseline respiratory component of the Sequential Organ Failure Assessment (SOFA) score (R-SOFA) ≥ 2 (indicative of severe respiratory failure), cardiovascular component of the SOFA score (CV-SOFA) ≥ 2 (indicative of shock), or R-SOFA ≥ 2 plus CV-SOFA ≥ 2 were compared by treatment arm. The efficacy endpoint of primary interest was 28-day all-cause mortality. Clinical response, time to death, and microbiologic response were also evaluated.

**Results:**

There were 726 participants in the intention-to-treat population; 633 with R-SOFA ≥ 2 (312 ceftolozane/tazobactam, 321 meropenem), 183 with CV-SOFA ≥ 2 (84 ceftolozane/tazobactam, 99 meropenem), and 160 with R-SOFA ≥ 2 plus CV-SOFA ≥ 2 (69 ceftolozane/tazobactam, 91 meropenem). Baseline characteristics, including causative pathogens, were generally similar in participants with R-SOFA ≥ 2 or CV-SOFA ≥ 2 across treatment arms. The 28-day all-cause mortality rate was 23.7% and 24.0% [difference: 0.3%, 95% confidence interval (CI) − 6.4, 6.9] for R-SOFA ≥ 2, 33.3% and 30.3% (difference: − 3.0%, 95% CI − 16.4, 10.3) for CV-SOFA ≥ 2, and 34.8% and 30.8% (difference: − 4.0%, 95% CI − 18.6, 10.3), respectively, for R-SOFA ≥ 2 plus CV-SOFA ≥ 2. Clinical cure rates were as follows: 55.8% and 54.2% (difference: 1.6%, 95% CI − 6.2, 9.3) for R-SOFA ≥ 2, 53.6% and 55.6% (difference: − 2.0%, 95% CI − 16.1, 12.2) for CV-SOFA ≥ 2, and 53.6% and 56.0% (difference: − 2.4%, 95% CI − 17.6, 12.8), respectively, for R-SOFA ≥ 2 plus CV-SOFA ≥ 2. Time to death was comparable in all SOFA groups across both treatment arms. A higher rate of microbiologic eradication/presumed eradication was observed for CV-SOFA ≥ 2 and R-SOFA ≥ 2 plus CV-SOFA ≥ 2 with ceftolozane/tazobactam compared to meropenem.

**Conclusions:**

The presence of severe respiratory failure or shock did not affect the relative efficacy of ceftolozane/tazobactam versus meropenem; either agent may be used to treat critically ill patients with vHABP/VABP.

*Trial registration*: ClinicalTrials.gov NCT02070757. Registered 25 February 2014, https://clinicaltrials.gov/ct2/show/NCT02070757

**Supplementary Information:**

The online version contains supplementary material available at 10.1186/s13613-022-01084-8.

## Background

Nosocomial pneumonia, comprising non-ventilated hospital-acquired, ventilated hospital-acquired, and ventilator-associated bacterial pneumonia (nvHABP/vHABP/VABP), is a relatively frequent infection in hospitalized patients, particularly those that are critically ill [1–4]. Mortality rates for nosocomial pneumonia range between approximately 20–50%, with the highest rates reported for vHABP and VABP [5–10]. Prompt initiation of appropriate antibacterial therapy improves survival; however, antibacterial resistance makes the selection of antibacterial agent(s) for the treatment of nosocomial pneumonia challenging [5, 9–13].

Ceftolozane/tazobactam is an approved combination antibacterial agent consisting of the antipseudomonal cephalosporin ceftolozane with the established β-lactamase inhibitor tazobactam. Ceftolozane/tazobactam has broad Gram-negative activity, that includes multidrug-resistant (MDR) *Pseudomonas aeruginosa* and extended-spectrum β-lactamase producing (ESBL+) Enterobacterales [14–16]. Ceftolozane/tazobactam penetrates the lung, and a dose of 3 g every 8 h provides target epithelial lining fluid (ELF) concentrations appropriate for nosocomial pneumonia [17–19]. The efficacy of ceftolozane/tazobactam for the treatment of vHABP/VABP was demonstrated in the phase 3, randomized, double-blind ASPECT-NP trial, in which participants received ceftolozane/tazobactam 3 g every 8 h or meropenem 1 g every 8 h, with dose adjustments for renal function [20].

Critically ill patients have altered antibacterial pharmacokinetics (PK) and pharmacodynamics (PD) [21], which may result in the inability to achieve optimal PK/PD targets for β-lactams, negatively impacting treatment outcomes [22, 23]. For these reasons, evaluation of treatment outcomes with ceftolozane/tazobactam specifically in critically ill patients with vHABP/VABP is important to determine its clinical utility in this high-risk population. We therefore conducted a post hoc subset analysis of efficacy outcomes in ASPECT-NP using respiratory and cardiovascular Sequential Organ Failure Assessment (SOFA) component scores as markers for greater severity of illness.

## Methods

### Study design overview

**Fig. 1:**
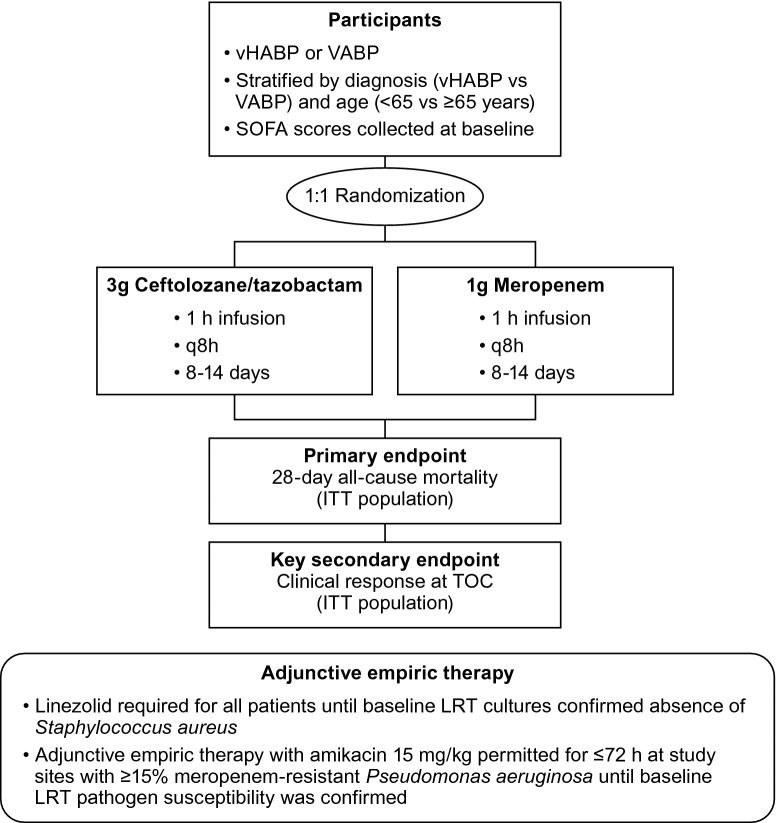
ASPECT-NP study design [20]. *h* hour, *ITT* intent-to-treat, *LRT* lower respiratory tract, *q8h* every 8 h, *TOC* test-of-cure, *SOFA* Sequential Organ Failure Assessment, *VABP* ventilator-associated bacterial pneumonia, *vHABP* ventilated hospital-acquired bacterial pneumonia

ASPECT-NP (protocol MK-7625A-008, ClinicalTrials.gov NCT02070757) was a phase 3, randomized, controlled, double-blind, multicenter, non-inferiority trial. Mechanically ventilated adults (≥ 18 years old) with nosocomial pneumonia were randomized 1:1, stratified by diagnosis (vHABP or VABP) and age (< 65 or ≥ 65 years old), to receive ceftolozane/tazobactam 3 g intravenously (IV) every 8 h or meropenem 1 g IV every 8 h, both adjusted for renal impairment. The study drugs were administered IV over 1 h for a duration of 8–14 days. Adjunctive linezolid 600 mg IV every 12 h (or a suitable alternative) was required for all participants until baseline lower respiratory tract (LRT) cultures confirmed the absence of *Staphylococcus aureus*. Adjunctive empirical therapy with amikacin 15 mg/kg IV daily was permitted for the first 72 h at study sites with ≥ 15% meropenem-resistant *P. aeruginosa*, as deemed necessary by the investigator (Fig. 1) [20].

A diagnosis of vHABP/VABP was based upon imaging showing new or progressive infiltrate, purulent tracheal secretions, and at least 1 additional clinical or laboratory criterion (fever, elevated/decreased white blood cell counts, or ≥ 15% immature neutrophils). vHABP was defined as pneumonia occurring in a mechanically ventilated participant in which at least one sign or symptom consistent with pneumonia (new/worsening cough, dyspnea, tachypnea, respiratory rate > 30 breaths per min, or hypoxemia) was present within the 24 h prior to intubation or within the 48 h after intubation in a participant who had been either hospitalized for ≥ 48 h or who had been discharged from a hospital within the prior 7 days (e.g., skilled nursing or other long-term care facility). VABP was defined as pneumonia in a participant who was mechanically ventilated for at least 48 h and had hypoxemia or required increased ventilator support. Quantitative LRT specimens were collected at baseline (within 36 h before the first dose of study drug) and post-baseline, including at the end-of-therapy (EOT) and at the test-of-cure (TOC) visit (7–14 days after the EOT visit) in participants who remained intubated or when clinically indicated. Pathogen identification and susceptibility were performed at local site microbiology laboratories and confirmed at a central microbiology laboratory, with standard broth microdilution methodology to verify minimum inhibitory concentrations (MICs) [20, 24].

Respiratory and cardiovascular SOFA component scores, along with total SOFA scores, were collected at baseline. A respiratory SOFA component score (R-SOFA) ≥ 2 corresponded to a PaO_2_/FiO_2_ < 300 mmHg. A cardiovascular SOFA component score (CV-SOFA) ≥ 2 corresponded to receipt of any dose of dopamine, dobutamine, epinephrine, or norepinephrine [25].

Participants were deemed clinically cured when they had a resolution of their baseline signs and symptoms, did not have any new signs or symptoms, and did not require additional antibacterial agents to treat their vHABP/VABP. Microbiologic response was categorized as eradication of all baseline pathogens [lower respiratory tract culture showing a ≥ 1-log reduction in baseline pathogenic bacterial burden, with a maximum per-pathogen count of 10^4^ colony-forming units (CFU) per mL for endotracheal aspirate specimens, 10^3^ CFU per mL for bronchoalveolar lavage specimens, and 10^2^ CFU per mL for protected brush specimens] or presumed eradication in cases with clinical cure but no respiratory material to culture at the EOT or TOC visit [20].

This study was conducted in accordance with principles of Good Clinical Practice and was approved by the appropriate institutional review boards and regulatory agencies for all sites. All participants (or legally acceptable representatives) provided informed consent. Complete details of the ASPECT-NP study have been published previously by Kollef et al. [20].

### Subset analysis

The population for this post hoc subset analysis consisted of all randomized participants from ASPECT-NP who had a baseline R-SOFA ≥ 2 or CV-SOFA ≥ 2, resulting in three intention-to-treat (ITT) groups: participants with R-SOFA ≥ 2, participants with CV-SOFA ≥ 2, and participants with R-SOFA ≥ 2 plus CV-SOFA ≥ 2. The microbiologic ITT (mITT) population included participants who received ≥ 1 dose of study drug and had ≥ 1 Gram-negative or streptococcal baseline LRT pathogen that was susceptible to at least one of the study drugs. Participants in the clinically evaluable (CE) population received study drug, adhered to the study protocol through the TOC visit, and had an evaluable clinical outcome at the TOC visit or clinical failure prior to the TOC visit.

Comparisons for outcomes between treatment arms in the R-SOFA ≥ 2, CV-SOFA ≥ 2, and R-SOFA ≥ 2 plus CV-SOFA ≥ 2 groups were based on a pre-specified analysis plan. Consistent with the pivotal study, the efficacy endpoint of primary interest was 28-day all-cause mortality. Clinical response, time to death, and microbiologic response were also evaluated in participants with R-SOFA ≥ 2, CV-SOFA ≥ 2, and R-SOFA ≥ 2 plus CV-SOFA ≥ 2. Additionally, to assess clinical utility in the most severely critically ill participants, outcomes between treatment arms were compared in participants with higher R- and CV-SOFA component scores (≥ 3 and 4) and SOFA > 6 versus ≤ 6.

The 28-day all-cause mortality, clinical response at the TOC visit, and microbiologic eradication/presumed eradication rates were compared using 2-sided unstratified Newcombe 95% confidence intervals (CIs) for the difference in proportions between the ceftolozane/tazobactam and meropenem arms according to R-SOFA, CV-SOFA, or SOFA [26]. Kaplan–Meier estimates were used to demonstrate time to death for each treatment arm in participants with a baseline R-SOFA ≥ 2, CV-SOFA ≥ 2, or R-SOFA ≥ 2 plus CV-SOFA ≥ 2.

## Results

**Fig. 2:**
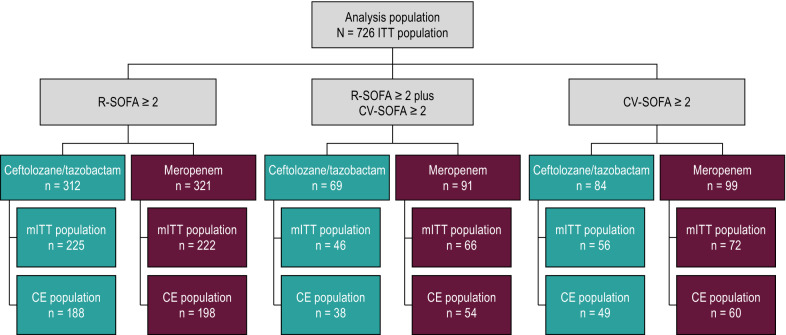
Participant and analysis population flow chart. *CE* clinically evaluable, *CV-SOFA* cardiovascular SOFA component score, *ITT* intention-to-treat, *LRT* lower respiratory tract, *mITT* microbiologic intention-to-treat, *R-SOFA* respiratory SOFA component score, *SOFA* Sequential Organ Failure Assessment

There were 312 participants in the ceftolozane/tazobactam arm and 321 participants in the meropenem arm who had a baseline R-SOFA ≥ 2, while there were 84 and 99 participants who had a baseline CV-SOFA ≥ 2 in each treatment arm, respectively. Sixty-nine ceftolozane/tazobactam-treated and 91 meropenem-treated participants had both R-SOFA ≥ 2 plus CV-SOFA ≥ 2 at baseline (Fig. 2).

Baseline and demographic characteristics were generally well balanced between treatment arms in participants with R-SOFA ≥ 2 or CV-SOFA ≥ 2, with some minor exceptions. In the CV-SOFA ≥ 2 group, there were more participants with vHABP and APACHE II score ≥ 20 in the ceftolozane/tazobactam than in the meropenem arm. A total SOFA score > 7 was more common in the meropenem arm in the R-SOFA ≥ 2 group, while failed prior antibacterial therapy was more frequent in the ceftolozane/tazobactam arm in the R-SOFA ≥ 2 and CV-SOFA ≥ 2 groups (Table 1).

**Table 1:** Baseline demographics and clinical characteristics of participants with a baseline R-SOFA ≥ 2 or CV-SOFA ≥ 2 (ITT population)

Variables	Respiratory SOFA component score ≥ 2		Cardiovascular SOFA component score ≥ 2	
	Ceftolozane/tazobactam (*n* = 312)	Meropenem (*n* = 321)	Ceftolozane/tazobactam (*n* = 84)	Meropenem (*n* = 99)
Primary diagnosis, *n* (%)				
vHABP	87 (27.9)	97 (30.2)	40 (47.6)	34 (34.3)
VABP	225 (72.1)	224 (69.8)	44 (52.4)	65 (65.7)
Age, years				
Median (range)	63 (18–98)	63 (18–92)	66 (24–88)	66 (18–87)
< 65 years, *n* (%)	171 (54.8)	178 (55.5)	37 (44.0)	46 (46.5)
≥ 65 years, *n* (%)	141 (45.2)	143 (44.5)	47 (56.0)	53 (53.5)
Sex at birth, *n* (%)				
Male	224 (71.8)	227 (70.7)	53 (63.1)	69 (69.7)
Female	88 (28.2)	94 (29.3)	31 (36.9)	30 (30.3)
Body mass index, kg/m^2^				
Median (range)	27.1 (15.1–49.3)	26.4 (15.5–67.2)	27.5 (15.1–49.3)	26.2 (15.6–56.0)
*n* missing	8	10	3	4
Race				
Asian	15 (4.8)	16 (5.0)	4 (4.8)	3 (3.0)
Black or African American	9 (2.9)	4 (1.2)	0	1 (1.0)
White	266 (85.3)	268 (83.5)	74 (88.1)	82 (82.8)
Other	4 (1.3)	7 (2.2)	2 (2.4)	2 (2.0)
Not reported or missing	18 (5.8)	26 (8.1)	4 (4.8)	11 (11.1)
CL_CR_ (mL/min)				
Median (range)	93.3 (16.0–336.2)	99.3 (15.7–401.0)^a^	74.5 (16.0–327.9)	92.0 (16.0–352.0)
≥ 150 (hyperclearance), *n* (%)	51 (16.3)	56 (17.4)	14 (16.7)	12 (12.1)
≥ 80 (normal), *n* (%)	196 (62.8)	212 (66.0)	41 (48.8)	61 (61.6)
> 50 to < 80 (mild impairment), *n* (%)	73 (23.4)	68 (21.2)	19 (22.6)	18 (18.2)
≥ 30 to ≤ 50 (moderate impairment), *n* (%)	29 (9.3)	23 (7.2)	16 (19.0)	10 (10.1)
≥ 15 to < 30 (severe impairment), *n* (%)	14 (4.5)	17 (5.3)	8 (9.5)	10 (10.1)
< 15 (ESRD), *n* (%)	0	0	0	0
APACHE II score				
Median (range)	17 (2–32)	17 (4–39)^a^	18 (8–33)	18 (6–37)
APACHE II score ≥ 20, *n* (%)	111 (35.6)	104 (32.5)^a^	34 (40.5)	32 (32.3)
Total SOFA score > 7, *n* (%)	93 (29.8)	120 (37.4)	66 (78.6)	82 (82.8)
CPIS > 8, *n* (%)	239 (76.6)	236 (73.5)	56 (66.7)	65 (65.7)
Duration of hospitalization prior to randomization, days				
Median (range)	8 (1–58)	7 (1–109)	8 (1–42)	6 (1–116)
*n* missing	2	2	1	1
Duration of MV prior to randomization, days				
Median (range)	4.8 (0–35.6)	4.7 (0–107.5)	3.1 (0–24.3)	3.9 (0–79.0)
*n* missing	0	2	0	0
Randomized while in the ICU, *n* (%)	294 (94.2)	297 (92.5)	70 (83.3)	93 (93.9)
Failed prior antibacterial(s) for vHABP/VABP, *n* (%)	45 (14.4)	31 (9.7)	15 (17.9)	6 (6.1)
Prior antibacterial therapy, *n* (%)	275 (88.1)	285 (88.8)	78 (92.9)	91 (91.9)
Bacteremia (any pathogen), *n* (%)	51 (16.3)	40 (12.5)	12 (14.3)	11 (11.1)
Gram-negative adjunctive therapy, *n* (%)	90 (28.8)	96 (29.9)^b^	33 (39.3)	36 (36.4)
Gram-positive adjunctive therapy, *n* (%)	303 (97.1)	309 (96.3)^a^	80 (95.2)	96 (97.0)

*APACHE* Acute Physiology and Chronic Health Evaluation, *CL*_*CR*_ creatinine clearance, *CPIS* clinical pulmonary infection score, *CV-SOFA* cardiovascular SOFA component score, *ESRD* end stage renal disease, *ICU* intensive care unit, *ITT* intention-to-treat, *MV* mechanical ventilation, *R-SOFA* respiratory SOFA component score, *SOFA* Sequential Organ Failure Assessment, *VABP* ventilator-associated bacterial pneumonia, *vHABP* ventilated hospital-acquired bacterial pneumonia

^a^*n* = 320

^b^*n* = 317

Nearly all baseline LRT pathogens across the R-SOFA ≥ 2 and the CV-SOFA ≥ 2 groups and treatment arms were Gram-negative bacteria. The most frequent causative pathogens in both groups were Enterobacterales (most often *Klebsiella pneumoniae* and *Escherichia coli*) and *P. aeruginosa*. Approximately a third of Enterobacterales in the R-SOFA ≥ 2 group carried ESBL genes, compared with about a quarter of Enterobacterales in the CV-SOFA ≥ 2 group (Table 2). The MIC_90_ for all Gram-negative pathogens in the R-SOFA ≥ 2 group was 16 mg/L for ceftolozane/tazobactam and 1 mg/L for meropenem. In the CV-SOFA ≥ 2 group, the MIC_90_ for all Gram-negative pathogens was 8 mg/L and 0.5 mg/L, respectively, in each treatment arm (Table 3).

**Table 2:** Baseline LRT pathogens in participants with a baseline R-SOFA ≥ 2 or CV-SOFA ≥ 2 (mITT population)

Variable	Respiratory SOFA component score ≥ 2		Cardiovascular SOFA component score ≥ 2	
	Ceftolozane/tazobactam (*n* = 225)	Meropenem (*n* = 222)	Ceftolozane/tazobactam (*n* = 56)	Meropenem (*n* = 72)
Number of baseline LRT pathogens				
Monomicrobial	138 (61.3)	142 (64.0)	36 (64.3)	45 (62.5)
Polymicrobial	87 (38.7)	80 (36.0)	20 (35.7)	27 (37.5)
Baseline LRT pathogens^a^				
Gram-negative	223 (99.1)	217 (97.7)	54 (96.4)	72 (100)
* Pseudomonas aeruginosa*	52 (23.1)	58 (26.1)	14 (25.0)	14 (19.4)
AmpC overexpressing	7 (3.1)	6 (2.7)	1 (1.8)	0
Enterobacterales	171 (76.0)	169 (76.1)	38 (67.9)	60 (83.3)
ESBL + Enterobacterales	72 (32.0)	67 (30.2)	14 (25.0)	17 (23.6)
* Citrobacter koseri*	5 (2.2)	7 (3.2)	1 (1.8)	3 (4.2)
* Enterobacter cloacae*	16 (7.1)	13 (5.9)	5 (8.9)	10 (13.9)
* Escherichia coli*	47 (20.9)	38 (17.1)	9 (16.1)	14 (19.4)
* Klebsiella aerogenes*	4 (1.8)	8 (3.6)	3 (5.4)	4 (5.6)
* Klebsiella oxytoca*	13 (5.8)	9 (4.1)	5 (8.9)	6 (8.3)
* Klebsiella pneumoniae*	79 (35.1)	86 (38.7)	16 (28.6)	24 (33.3)
* Proteus mirabilis*	21 (9.3)	19 (8.6)	3 (5.4)	6 (8.3)
* Serratia marcescens*	11 (4.9)	12 (5.4)	6 (10.7)	3 (4.2)
* Acinetobacter baumannii*	18 (8.0)	12 (5.4)	3 (5.4)	1 (1.4)
* Haemophilus influenzae*	18 (8.0)	16 (7.2)	5 (8.9)	8 (11.1)
Gram-positive	9 (4.0)	15 (6.8)	3 (5.4)	3 (4.2)
* Streptococcus pneumoniae*	4 (1.8)	7 (3.2)	1 (1.8)	2 (2.8)

Data are presented as count (percentage)

Gram-negative pathogens with *n* ≥ 10 within organism species pooled across both treatment arms for either SOFA component score were included. ESBL + Enterobacterales were reported collectively. All Gram-positive pathogens were included

*CV-SOFA* cardiovascular SOFA component score, *ESBL* + extended-spectrum β-lactamase producing, *LRT* lower respiratory tract, *mITT* microbiologic intention-to-treat, *R-SOFA* respiratory SOFA component score, *SOFA* Sequential Organ Failure Assessment

^a^Participants with more than 1 pathogen isolated at baseline were counted only once within each pathogen category (e.g., Gram-negative or Gram-positive), once within organism/group (e.g., Enterobacterales) and once for each pathogen (e.g., *E. coli*)

**Table 3:** MIC summary of baseline LRT pathogens in participants with a baseline R-SOFA ≥ 2 or CV-SOFA ≥ 2 (mITT population)

Pathogen	Respiratory SOFA component score ≥ 2		Cardiovascular SOFA component score ≥ 2	
	Ceftolozane/tazobactam (*n* = 222)	Meropenem (*n* = 219)	Ceftolozane/tazobactam (*n* = 53)	Meropenem (*n* = 72)
All Gram-negatives				
*n*1	312	307	75	106
MIC range	< 0.064 to ≥ 256	< 0.064 to ≥ 256	< 0.064 to ≥ 256	< 0.064 to 128
MIC_50_	0.5	< 0.064	0.5	< 0.064
MIC_90_	16	1	8	0.5
* Pseudomonas aeruginosa*				
*n*1	52	58	14	14
MIC range	0.25 to ≥ 256	< 0.064 to 16	0.25 to ≥ 256	0.125 to 2
MIC_50_	1	0.5	1	0.5
MIC_90_	4	8	2	1
AmpC overexpressing				
*n*1	7	6	1	0
MIC range	1 to 8	0.25 to 16	2 to 2	N/a
MIC_50_	2	8	2	N/a
MIC_90_	8	16	2	N/a
Enterobacterales				
*n*1	206	206	47	79
MIC range	0.125 to ≥ 256	< 0.064 to 16	0.125 to 128	< 0.064 to 0.25
MIC_50_	0.5	< 0.064	0.5	< 0.064
MIC_90_	16	0.25	16	< 0.064
ESBL + Enterobacterales				
*n*1	77	74	15	19
MIC range	0.25 to ≥ 256	< 0.064 to 1	0.25 to 128	< 0.064 to < 0.064
MIC_50_	4	< 0.064	4	< 0.064
MIC_90_	128	1	64	< 0.064
* Citrobacter koseri*				
*n*1	5	7	1	3
MIC range	0.125 to 1	< 0.064 to 0.25	0.125 to 0.125	< 0.064 to 0.25
MIC_50_	0.25	< 0.064	0.125	< 0.064
MIC_90_	1	0.25	0.125	0.25
* Enterobacter cloacae*				
*n*1	16	13	5	10
MIC range	0.125 to 8	< 0.064 to < 0.064	0.25 to 8	< 0.064 to < 0.064
MIC_50_	0.5	< 0.064	1	< 0.064
MIC_90_	8	< 0.064	8	< 0.064
* Escherichia coli*				
*n*1	47	38	9	14
MIC range	0.125 to 4	< 0.064 to 0.125	0.125 to 0.5	< 0.064 to < 0.064
MIC_50_	0.25	< 0.064	0.25	< 0.064
MIC_90_	1	< 0.064	0.5	< 0.064
* Klebsiella aerogenes*				
*n*1	3	7	2	4
MIC range	0.125 to 0.25	< 0.064 to 1	0.125 to 0.25	< 0.064 to 0.25
MIC_50_	0.125	0.25	0.125	0.125
MIC_90_	0.25	1	0.25	0.25
* Klebsiella oxytoca*				
*n*1	13	9	5	6
MIC range	0.125 to 2	< 0.064 to < 0.064	0.125 to 0.25	< 0.064 to < 0.064
MIC_50_	0.25	< 0.064	0.25	< 0.064
MIC_90_	0.25	< 0.064	0.25	< 0.064
* Klebsiella pneumonia*				
*n*1	77	86	16	24
MIC range	0.25 to ≥ 256	< 0.064 to 16	0.25 to 128	< 0.064 to < 0.064
MIC_50_	2	< 0.064	0.5	< 0.064
MIC_90_	128	1	64	< 0.064
* Proteus mirabilis*				
*n*1	21	19	3	6
MIC range	0.25 to 8	< 0.064 to 0.125	0.25 to 0.5	< 0.064 to 0.125
MIC_50_	0.5	< 0.064	0.5	< 0.064
MIC_90_	4	0.125	0.5	0.125
* Serratia marcescens*				
* n*1	10	12	5	3
MIC range	0.5 to 4	< 0.064 to 0.125	0.5 to 8	< 0.064 to < 0.064
MIC_50_	0.5	< 0.064	2	< 0.064
MIC_90_	2	< 0.064	8	< 0.064
* Acinetobacter baumannii*				
*n*1	18	12	3	1
MIC range	< 0.064 to ≥ 256	0.25 to ≥ 256	< 0.064 to 8	0.25 to 0.25
MIC_50_	8	1	4	0.25
MIC_90_	≥ 256	128	8	0.25
* Haemophilus influenzae*				
*n*1	18	15	5	8
MIC range	< 0.064 to 0.5	< 0.064 to 0.125	< 0.064 to 0.5	< 0.064 to 0.125
MIC_50_	0.125	< 0.064	0.125	< 0.064
MIC_90_	0.25	0.125	0.5	0.125
All Gram-positive				
*n*1	8	14	2	3
MIC range	< 0.064 to 32	< 0.064 to 1	0.25 to 4	< 0.064 to 1
MIC_50_	0.25	< 0.064	0.25	< 0.064
MIC_90_	32	1	4	1
* Streptococcus pneumoniae*				
*n*1	4	7	1	2
MIC range	< 0.064 to 0.25	< 0.064 to 1	0.25 to 0.25	< 0.064 to < 0.064
MIC_50_	0.064	< 0.064	0.25	< 0.064
MIC_90_	0.25	1	0.25	< 0.064

*n* is the number of participants in the population

*n*1 is the number of pathogens with baseline MIC data available

MIC is reported in mg/L and was measured by broth microdilution testing

MIC_50_ is the minimum inhibitory concentration required to inhibit the growth of 50% of the pathogens

MIC_90_ is the minimum inhibitory concentration required to inhibit the growth of 90% of the pathogens

Pathogens with *n* ≥ 10 within organism species pooled across both treatment arms for any SOFA component score. ESBL + Enterobacterales reported collectively

Each pathogen was counted once per participant and where there were multiple values, the pathogen with the highest MIC was used

*CV-SOFA* cardiovascular SOFA component score, *ESBL* + extended-spectrum β-lactamase producing, *LRT* lower respiratory tract, *MIC* minimum inhibitory concentration, *mITT* microbiologic intention-to-treat, *R-SOFA* respiratory SOFA component score, *SOFA* Sequential Organ Failure Assessment

**Fig. 3:**
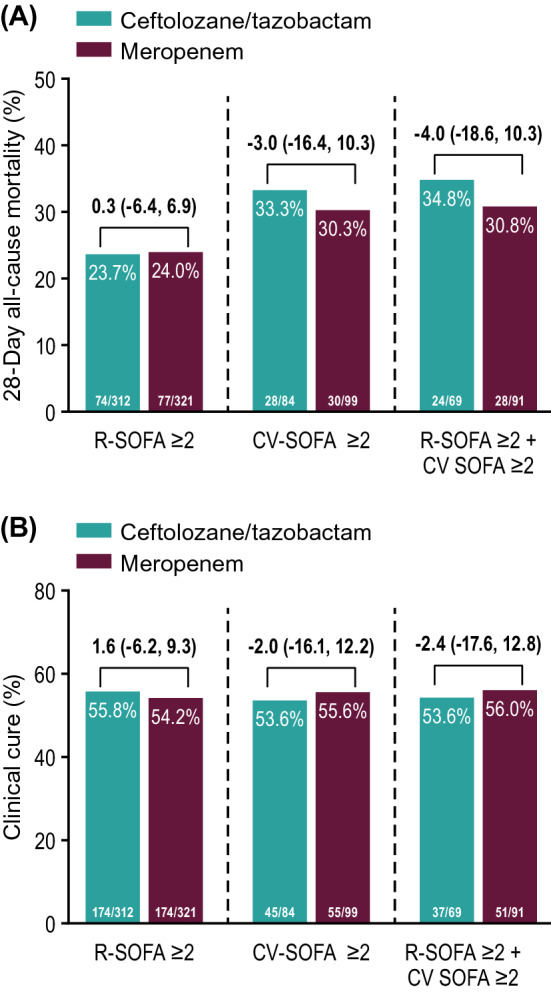
Mortality (upper panel **A**) and cure at TOC (bottom panel **B**) by SOFA component score (ITT population). Treatment differences were calculated as unstratified Newcombe 95% CIs; positive differences are in favor of ceftolozane/tazobactam, negative differences are in favor of meropenem. Participants whose 28-day mortality outcome was missing or unknown were analyzed as deceased. Participants with clinical failure at the EOT visit were counted as failures at the TOC visit. *CI* confidence interval, *CV-SOFA* cardiovascular, *EOT* end-of-therapy, *R-SOFA* respiratory SOFA component score, *SOFA* Sequential Organ Failure Assessment, *TOC* test-of-cure

**Fig. 4:**
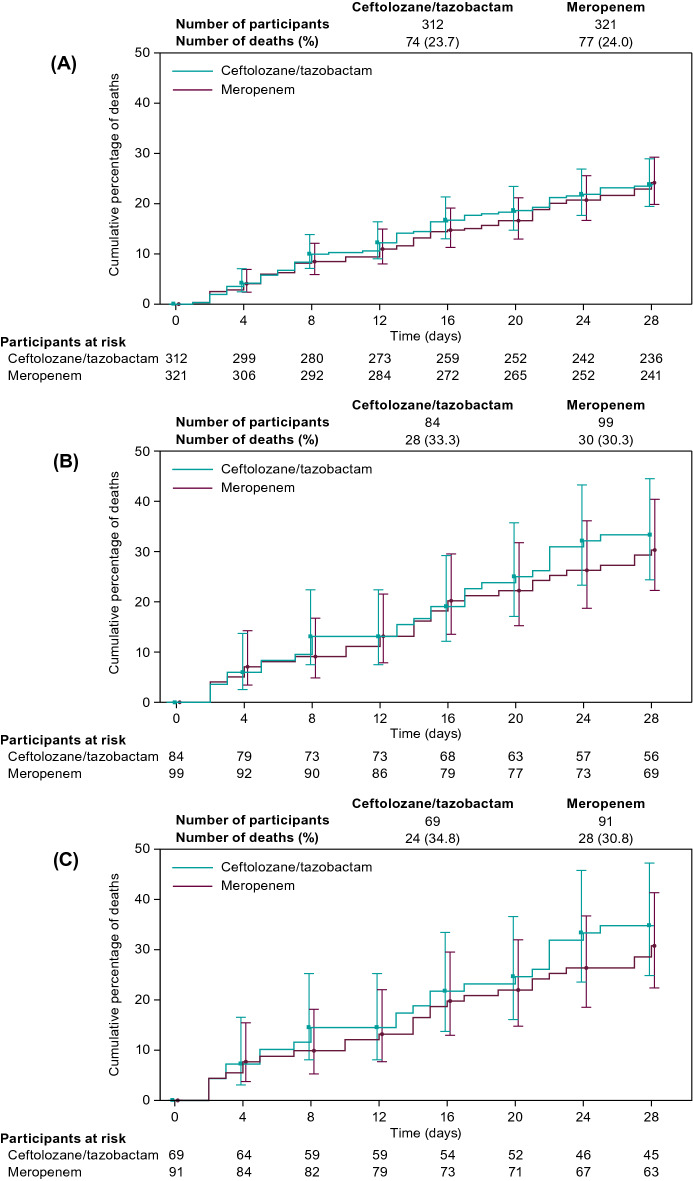
Time to death by SOFA component score (ITT population). R-SOFA ≥ 2 (upper panel **A**), CV-SOFA ≥ 2 (middle panel **B**), R-SOFA ≥ 2 plus CV-SOFA ≥ 2 (bottom panel **C**). *CV-SOFA* cardiovascular SOFA component score, *R-SOFA* respiratory SOFA component score, *SOFA* Sequential Organ Failure Assessment

Although the observed mortality in the CV-SOFA ≥ 2 and R-SOFA ≥ 2 plus CV-SOFA ≥ 2 groups was higher compared to the mortality in the R-SOFA ≥ 2 group, the 28-day all-cause mortality rates across treatment arms were comparable within each individual SOFA group in the ITT population (Fig. 3a). Clinical cure rates at TOC with ceftolozane/tazobactam and meropenem were unaffected by R-SOFA ≥ 2, CV-SOFA ≥ 2, or R-SOFA ≥ 2 plus CV-SOFA ≥ 2 in the ITT (Fig. 3b) and CE population (Additional file 1: Fig. S1). There was no observed difference between ceftolozane/tazobactam and meropenem in time to death in participants with a baseline R-SOFA ≥ 2, CV-SOFA ≥ 2, or R-SOFA ≥ 2 plus CV-SOFA ≥ 2 in the ITT population (Fig. 4a–c).

**Fig. 5:**
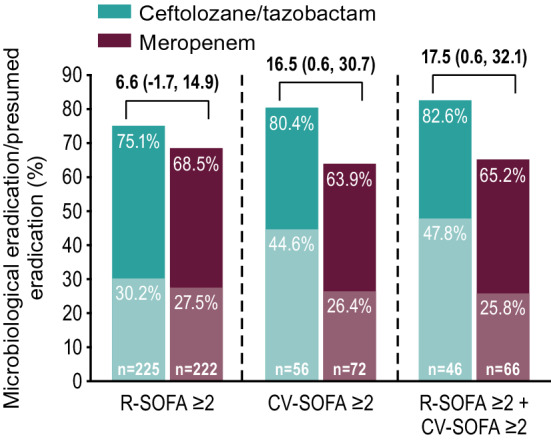
Microbiologic eradication/presumed eradication at TOC by SOFA component score (mITT population). Observed microbiologic eradication is represented by the lighter colored bars; presumed eradication is represented by the darker colored bars. Treatment differences were calculated as unstratified Newcombe 95% CIs; positive differences are in favor of ceftolozane/tazobactam; negative differences are in favor of meropenem. Participants with missing culture and clinical responses that were failure, indeterminate, or missing were counted as presumed failures. *CI* confidence interval, *CV-SOFA* cardiovascular SOFA component score, *R-SOFA* respiratory SOFA component score, *SOFA* Sequential Organ Failure Assessment, *TOC* test-of-cure

Microbiologic eradication/presumed eradication rates were higher in ceftolozane/tazobactam-treated participants with CV-SOFA ≥ 2 and R-SOFA ≥ 2 plus CV-SOFA ≥ 2 than with a baseline R-SOFA ≥ 2, while the microbiologic eradication/presumed eradication rates were consistent across all three SOFA groups in meropenem-treated participants. Additionally, a higher percentage of ceftolozane/tazobactam-treated participants had microbiologic eradication/presumed eradication as compared to meropenem-treated participants in the CV-SOFA ≥ 2 and R-SOFA ≥ 2 plus CV-SOFA ≥ 2 groups in the mITT population (Fig. 5).

Mortality and clinical cure rates were comparable across treatment arms in the higher SOFA component score (Additional file 1: Fig. S2) and SOFA groups (Additional file 1: Fig. S3) in the ITT population, with higher mortality rates observed in the R-SOFA 4, CV-SOFA ≥ 3, CV-SOFA 4, and SOFA > 6 groups, and clinical cure rates ranging between approximately 50% to 60% in all SOFA component score and SOFA groups. Microbiologic eradication/presumed eradication rates were similar across treatment arms in the SOFA analysis in the mITT population (Additional file 1: Fig. S3).

## Discussion

Our subset analysis in critically ill participants with vHABP/VABP from the ASPECT-NP trial found that 28-day all-cause mortality and clinical cure rates at the TOC visit were comparable between ceftolozane/tazobactam and meropenem regardless of R-SOFA ≥ 2, CV-SOFA ≥ 2, or R-SOFA ≥ 2 plus CV-SOFA ≥ 2. The 28-day all-cause mortality rates in both treatment arms relative to the overall ITT population in the ASPECT-NP trial [24.0% (87/362) and 25.3% (92/364), respectively] [20] were, as expected, worse in participants with CV-SOFA ≥ 2 and R-SOFA ≥ 2 plus CV-SOFA ≥ 2. However, the 95% CI for the differences in mortality rates between the treatment arms included zero in all SOFA groups analyzed. Across all three SOFA groups, clinical cure rates in both treatment arms remained unchanged and aligned with the rates in ASPECT-NP [54.4% (197/362) and 53.3% (194/364), respectively] [20]. Time to death did not differ between ceftolozane/tazobactam and meropenem in participants with R-SOFA ≥ 2, CV-SOFA ≥ 2, or R-SOFA ≥ 2 plus CV-SOFA ≥ 2.

No difference was seen between ceftolozane/tazobactam and meropenem in microbiologic response rate in the mITT population with R-SOFA ≥ 2. A higher rate of microbiologic eradication/presumed eradication, driven by documented microbiologic eradication, was observed with ceftolozane/tazobactam in participants with CV-SOFA ≥ 2 and R-SOFA ≥ 2 plus CV-SOFA ≥ 2. This could be related to the optimized ceftolozane/tazobactam dose of 3 g every 8 h infused over 1 h, which has been shown to provide mean ELF penetration ratios of 0.5 and 0.6 for each component, respectively, in mechanically ventilated adults with pneumonia [17]. This may have increased the probability of reaching target ELF exposures with ceftolozane/tazobactam. Meropenem produced lower mean ELF penetration ratios in a similar population, even with higher doses and prolonged infusion times [27]. Although it has been suggested that higher doses of meropenem may be beneficial in this patient population [28], the MIC_90_ was 1 mg/L for *P. aeruginosa* and < 0.064 mg/L for Enterobacterales in the CV-SOFA ≥ 2 group. In addition, efficacy outcomes in meropenem-treated participants with augmented renal clearance at baseline were comparable to those with normal renal function in Shorr et al. [29]. Altogether this suggests the meropenem dose was adequate; however, given the complex PK in critically ill patients, we cannot confirm whether the meropenem dose or infusion time influenced the microbiologic eradication rates observed in this analysis.

A strength of this study is that the ASPECT-NP trial enrolled exclusively ventilated participants, all of whom were critically ill, with a vast majority admitted to the ICU. This is reflected by the high prevalence of severe respiratory failure or shock in this subset analysis. SOFA scores were collected prospectively from all participants in the ASPECT-NP trial. This provided a large, randomized sample of participants with vHABP/VABP and elevated SOFA component scores for our analysis. The baseline LRT pathogens identified in the participants were representative of the pathogens associated with nosocomial pneumonia. Moreover, outcomes in participants with higher SOFA component scores, which included participants with PaO_2_/FiO_2_ < 200 mmHg or vasopressor-requiring shock, aligned with those observed for R- and CV-SOFA ≥ 2. For example, the observed mortality rates with CV-SOFA ≥ 3 (ceftolozane/tazobactam 35.1%, meropenem 30.4%) were similar to the rates with CV-SOFA ≥ 2 (ceftolozane/tazobactam 33.3%, meropenem 30.3%).

One limitation of this study is that we used individual components of the SOFA score to define the presence of severe respiratory failure (as a baseline R-SOFA ≥ 2) and the presence of shock (as a baseline CV-SOFA ≥ 2). Although the SOFA score is a well-validated scoring system to evaluate the degree of organ dysfunction in critically ill patients [30] and individual organ system components of the score predict mortality [31], individual organ system component scores are not routinely used as unique scores to measure respiratory or cardiovascular dysfunction. The results of the SOFA analysis generally supported the outcomes of the individual SOFA component score analyses. Mortality rates were higher with SOFA > 6 (ceftolozane/tazobactam 31.6%, meropenem 29.9%) as compared to SOFA ≤ 6 and corresponded with the morality rates observed with CV-SOFA ≥ 2 (ceftolozane/tazobactam 33.3%, meropenem 30.3%). Clinical cure rates remained within the 50% to 60% range as in the SOFA component score analyses. However, no differences were identified between treatment arms for microbiologic eradication/presumed eradication based on SOFA, unlike the observations based on CV-SOFA ≥ 2 and R-SOFA ≥ 2 plus CV-SOFA ≥ 2. The results of this study cannot be applied to critically ill patients who are immunocompromised, have received an organ or stem cell transplant, have blood dyscrasias, or have cystic fibrosis, as they were excluded from the ASPECT-NP trial; yet these may be co-morbidities in patients with nosocomial pneumonia [32–34]. Lastly, the comparisons between outcomes were not powered for non-inferiority testing or controlled for multiplicity. Given the small sample sizes in some of the subsets, the corresponding results must be interpreted with caution.

In summary, ceftolozane/tazobactam was an effective treatment for vHABP/VABP in adults with organ failure, regardless of degree of severity of illness. Greater severity of illness did not affect the relative efficacy of ceftolozane/tazobactam versus meropenem and either agent is an appropriate option for the treatment of vHABP/VABP caused by susceptible Gram-negative pathogens.

## Supplementary Information

**Additional file 1. Fig. S1**: Clinical cure at TOC by SOFA component score group (CE population). **Fig. S2**: Mortality (upper panel A) and clinical cure at TOC (bottom panel B) by SOFA component score ≥ 3 and 4 (ITT population). **Fig. S3**: Outcomes by SOFA ≤ 6 and > 6.

## Data Availability

The data sharing policy, including restrictions, of Merck Sharp & Dohme LLC, a subsidiary of Merck & Co., Inc., Rahway, NJ, USA (MSD) is available at http://engagezone.msd.com/ds_documentation.php. Requests for access to the study data can be submitted through the EngageZone site or via email to dataaccess@merck.com.

## Abbreviations

APACHE: Acute Physiology and Chronic Health Evaluation
CE: Clinically evaluable
CFU: Colony-forming units
CI: Confidence intervals
CL_CR_: Creatinine clearance
CPIS: Clinical pulmonary infection score
CV-SOFA: Cardiovascular SOFA component score
ELF: Epithelial lining fluid
EOT: End-of-therapy
ESBL+: Extended-spectrum β-lactamase producing
h: Hour
ICU: Intensive care unit
ITT: Intention-to-treat
IV: Intravenously
LRT: Lower respiratory tract
MDR: Multidrug-resistant
mITT: Microbiologic intention-to-treat
MV: Mechanical ventilation
nvHABP: Non-ventilated hospital-acquired pneumonia
PD: Pharmacodynamics
PK: Pharmacokinetics
q8h: Every 8 h
R-SOFA: Respiratory SOFA component score
SOFA: Sequential Organ Failure Assessment
TOC: Test-of-cure
VABP: Ventilator-associated bacterial pneumonia
vHABP: Ventilated hospital-acquired pneumonia
