# Supplementary material for: Outcomes in participants with ventilated nosocomial pneumonia and organ failure treated with ceftolozane/tazobactam versus meropenem: a subset analysis of the phase 3, randomized, controlled ASPECT-NP trial
Source: Ann Intensive Care. 2023 Feb 11;13:8. doi: 10.1186/s13613-022-01084-8 (PMC9922343; doi:10.1186/s13613-022-01084-8)
Supplement: Supplementary file 1 — Additional file 1. Fig. S1: Clinical cure at TOC by SOFA component score group (CE population). Fig. S2: Mortality (upper panel A) and clinical cure at TOC (bottom panel B) by SOFA component score ≥ 3 and 4 (ITT population). Fig. S3: Outcomes by SOFA ≤ 6 and > 6. [file 13613_2022_1084_MOESM1_ESM.docx]

**Outcomes in participants with ventilated nosocomial pneumonia and organ failure treated with ceftolozane/tazobactam versus meropenem
– subset analysis of the phase 3 randomized, controlled ASPECT-NP trial**

Ignacio Martin-Loeches, MD, et al.

**Supplement**

**
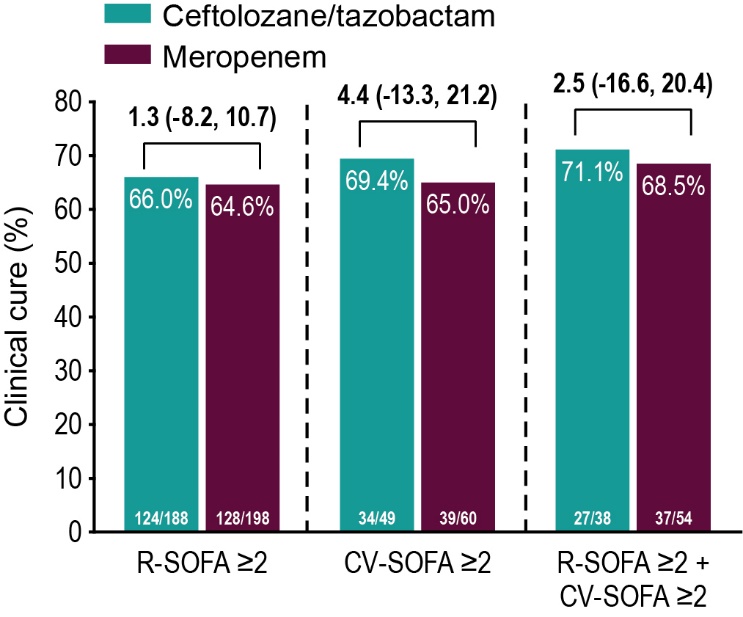
**

**Fig. S1.** Clinical cure at TOC by SOFA component score (CE population). Treatment differences were calculated as unstratified Newcombe 95% CIs; positive differences are in favor of ceftolozane/tazobactam, negative differences are in favor of meropenem. Participants with clinical failure at the EOT visit were counted as failures at the TOC visit. *CE* clinically evaluable, *CI* confidence interval, *CV-SOFA* cardiovascular, *EOT* end-of-therapy, *R-SOFA* respiratory SOFA component score, *SOFA* Sequential Organ Failure Assessment, *TOC* test-of-cure


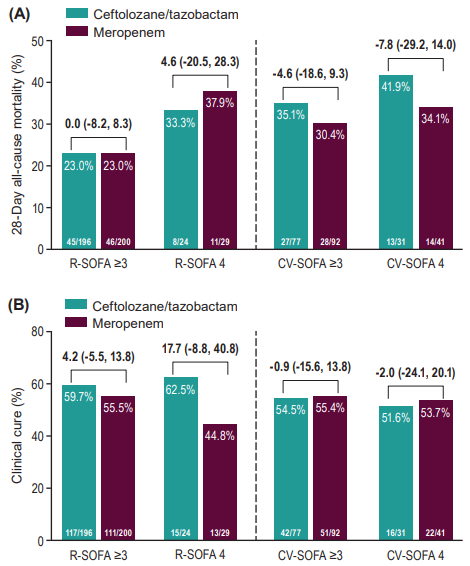


**Fig. S2.** Mortality (upper panel A) and clinical cure at TOC (bottom panel B) by SOFA component score ≥3 and 4 (ITT population). Treatment differences were calculated as unstratified Newcombe 95% CIs; positive differences are in favor of ceftolozane/tazobactam, negative differences are in favor of meropenem. Participants whose 28-day mortality outcome was missing or unknown were analyzed as deceased**.** Participants with clinical failure at the EOT visit were counted as failures at the TOC visit. *CI* confidence interval, *CV-SOFA* cardiovascular SOFA component score, *EOT* end-of-therapy, *R-SOFA* respiratory SOFA component score, *SOFA* Sequential Organ Failure Assessment, *TOC* test-of-cure


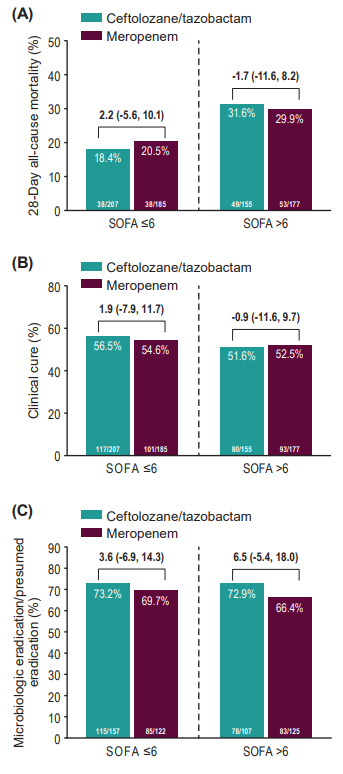


**Fig. S3.** Outcomes by SOFA ≤6 and >6. Mortality (ITT population) (upper panel A), clinical cure at TOC (ITT population) (middle panel B), microbiologic eradication at TOC (mITT population) (bottom panel C). Treatment differences were calculated as unstratified Newcombe 95% CIs; positive differences are in favor of ceftolozane/tazobactam, negative differences are in favor of meropenem. Participants whose 28-day mortality outcome was missing or unknown were analyzed as deceased. Participants with clinical failure at the EOT visit were counted as failures at the TOC visit. Participants with missing culture and clinical responses that were failure, indeterminate, or missing were counted as microbiologic failures. *CI* confidence interval, *EOT* end-of-therapy, *SOFA* Sequential Organ Failure Assessment, *TOC* test-of-cure
